# Editorial: Translational research in thyroid cancer, Volume II

**DOI:** 10.3389/fendo.2023.1223976

**Published:** 2023-06-13

**Authors:** Sheue-yann Cheng, Vasyl Vasko, Kirk Ernest Jensen

**Affiliations:** ^1^ Laboratory of Molecular Biology, National Cancer Institute, Bethesda, MD, United States; ^2^ Department of Pediatrics, Uniformed Services University of the Health Sciences, Bethesda, MD, United States

**Keywords:** thyroid cancer, radiation, oncogenes, microenvironment, molecular diagnosis

Translational thyroid cancer research moves forward the diagnosis and treatment of thyroid cancer by fostering interdisciplinary investigations and efforts of basic and clinical scientists. This Research Topic, “*Translational Research in Thyroid Cancer, Volume II*” contains a collection of studies that encompass a range of scientific findings addressing radiation-induced thyroid cancers, the biology of the thyroid cancer microenvironment, and thyroid cancer diagnosis.

Epidemiological studies have shown a worldwide increase in thyroid cancer incidence in recent decades. This increase is largely due to more frequent detection of papillary thyroid carcinoma, in particularly, papillary thyroid microcarcinomas (1). Previously reported data has demonstrated histopathological differences between radiogenic and sporadic PTCs, and has shown more aggressive behavior of radiogenic tumors, most notably in children and adolescents. Bogdanova et al. undertook a retrospective analysis of microcarcinomas (MPTCs) from both individuals with and without a history of exposure to ionizing radiation. The authors characterized invasive properties, clinical data and recurrence rate in radiogenic and sporadic MPTCs. Results of this study demonstrated that irradiation did not affect tumor phenotype, was not associated with more pronounced invasive properties, and did not worsen prognosis in pediatric or young adult patients with MPTC, thus implying that a history of radiation may not be a pivotal factor for determining treatment strategy in such patients.

Exposure to ionizing radiation is a risk factor for the development of thyroid cancer (2). More than 6,000 cases of thyroid cancer have been detected in individuals who were children or adolescents living in the immediate surrounding area at the time of the Chernobyl accident (3). Excess cases of thyroid cancer in exposed children were first detected after the accident in 1990. Such cases are continuing to accumulate, albeit at a much slower rate, now more than 30 years later (4, 5). Bogdanova et al. examined the impact of temporal latency from the time of radiation exposure to MPTC diagnosis. Specifically, the authors have addressed whether clinicopathological characteristics and prognosis of post-Chernobyl MPTCs were changing with regard to the: i) latency period, ii) probability of causation (POC) of a tumor due to radiation, and iii) tumor size. Further, the authors analyzed the relationships between the presence of a BRAF mutation and response to radioiodine treatment in patients with radiogenic PTCs. This study demonstrated that latency period was significantly associated with the reduction of POC level, tumor size and the frequency of fully encapsulated MPTCs. A longer latency period led to an increase in the frequency of the BRAFV600E mutation, oncocytic changes, and a decrease in the probability of excellent response to treatment with radioactive iodine.

The BRAF mutation has been shown to be associated with aggressive clinical behavior in patients with PTCs, and growing evidence demonstrates that the tumor microenvironment plays a role in cancer progression (6). Currently, there is limited information on how the BRAF mutation can affect the tumor immune microenvironment (TIME) in thyroid cancer, including interactions between neoplastic thyroid follicular cells and extra-cellular matrix components. Xia et al., analyzed the mRNA-seq data and corresponding clinical data of PTC patients, and calculated the TIME scores, providing a comprehensive TIME-related prognostic model to increase the predictive accuracy of progression-free survival (PFS) in patients with BRAF-mutated PTC. Another study in this collection also address the role of microenvironment with specific focus on cell-to-cell communication in thyroid cancer. Cell-to-cell communication based on ligand-receptor interactions plays a role in tumor progression and resistance to therapies. Hui He et al. have employed a single-cell RNA sequencing (scRNA-seq) to investigate cellular functionalities of DNA and RNA in different cellular subsets. The authors screened the critical ligand-receptor pair: CADM1_CADM1 by analyzing scRNA-seq data from human PTC samples and bulk RNA-seq data. This paper also discusses the potential utility of therapies targeting ligand-receptor pairs in the TIME in patients with aggressive thyroid cancer.

Genetic alterations can be detected in fine-needle aspiration (FNA) material taken from thyroid nodules. Molecular testing has helped to improve cancer diagnosis and management of patients in many cases. This testing has been most notably leveraged in patients with indeterminate results on FNA.Whitmer et al. investigated the epidemiology of thyroid stimulating hormone receptor (TSHR) variants in indeterminate (INT) FNAs submitted for molecular analysis by the Afirma Genomic Sequencing Classifier (GSC) testing. The authors also assessed the malignancy rate in GSC-S ITN with TSHR variants, representing the largest such cohort published to date. Population based risk assessments of thyroid nodule mutations provide beneficial guidance for a general approach to patients with ITN and thyroid cancer. However, physicians treat individuals, and this study demonstrates the utility of the Afirma GSC to risk stratify patients with TSHR mutated ITN in a clinically meaningful way.

The current risk adapted approach to thyroid cancer management aligns the intensity of therapeutic interventions, including surgical treatment, subsequent RAI therapy, and thyrotropin (TSH) suppressive therapy, with clinical outcome risks. This typically results in more aggressive interventions for high-risk patients and less aggressive therapies for low-risk patients (7). Unfortunately, there is no guarantee that more aggressive therapies will necessarily improve clinical outcomes in high-risk patients or conversely that more minimalistic therapies would necessarily be effective for low-risk patients. Jiang et al. examined the effectiveness of repeated 131 I therapy for radioiodine-avid lymph nodes from differentiated thyroid cancer on the initial post-therapy scan. The authors analyzed data from 170 patients with differentiated thyroid cancer presenting with 131I positive lymph nodes on the initial post-therapy scan. Their results show that approximately one-quarter of patients with 131I positive lymph nodes on initial PTS, remain stable after one cycle of 131I therapy and do not need repeated therapy.

The understanding of thyroid cancer biology is constantly evolving. Development of novel treatment approaches requires an interdisciplinary approach integrating multiple scientific and clinical disciplines.

This Research Topic has expanded our current understanding of radiation-inducible thyroid cancers, thyroid tumor microenvironment, and provides additional information regarding the diagnostic effectiveness of molecular testing in risk stratification of patients with thyroid nodules.

## Author contributions

All authors listed have made a substantial, direct, and intellectual contribution to the work and approved it for publication.
